# The patient perspective on sirolimus for epithelioid hemangioendothelioma (EHE): results of a community survey highlighting the importance of equitable access to treatments

**DOI:** 10.3389/fonc.2024.1367237

**Published:** 2024-02-26

**Authors:** Denise Robinson, Hugh Leonard, Giacomo Giulio Baldi, William D. Tap, Robin L. Jones, Silvia Stacchiotti, Pan Pantziarka

**Affiliations:** ^1^The EHE Foundation, Hobart, WI, United States; ^2^EHE Rare Cancer Charity UK, Kingston-Upon-Thames, United Kingdom; ^3^Department of Oncology, Hospital of Prato, Azienda USL Toscana Centro, Prato, Italy; ^4^Department of Medicine, Memorial Sloan Kettering Cancer Center and Weill Cornell Medical College, New York, NY, United States; ^5^Sarcoma Unit, The Royal Marsden Hospital and The Institute of Cancer Research, London, United Kingdom; ^6^Department of Medical Oncology, Fondazione IRCCS Istituto Nazionale dei Tumori, Milan, Italy; ^7^Anticancer Fund, Meise, Belgium; ^8^George Pantziarka TP53 Trust, London, United Kingdom

**Keywords:** sarcoma, epithelioid hemangioendothelioma, sirolimus, patient advocates, drug repurposing, patient survey, EHE

## Abstract

**Background:**

Epithelioid hemangioendothelioma (EHE) is an ultra-rare, vascular sarcoma with clinical presentation ranging from an indolent to an aggressive form. Over 50% of patients present with metastatic disease, requiring systemic therapy, although no systemic therapies are specifically approved for EHE. Retrospective evidence supports the activity of mTOR inhibitors (e.g. sirolimus), although available only off-label. EHE patients and advocates are therefore working to support approval of effective treatments by collecting data on patient perspectives and experiences.

**Materials and methods:**

In February 2023, the EHE Rare Cancer Charity (UK) and The EHE Foundation (US), with other advocates, conducted a survey of perspectives and experiences of EHE patients regarding the use and accessibility of sirolimus. The survey consisted of 20 questions designed for individuals undergoing treatment, those who had been treated, or had never been treated with the drug. Widely promoted within the patient community, the online survey categorized patients into three cohorts for the analysis: liver transplant patients, non-transplant patients who had ever taken sirolimus and sirolimus-naïve non-transplant patients.

**Results:**

The survey evaluated data from 129 patient responses from 21 countries, mostly from USA, UK, Australia, and Canada (70%). The liver transplant, sirolimus and non-sirolimus cohorts were 16%, 25% and 59%, respectively. In the sirolimus group 66% reported treatment durations exceeding one year, with 16% exceeding five years, indicating the drug’s efficacy. In the non-sirolimus group, the drug was not available for 42% and for 11% sirolimus was available but not selected for treatment because of its off-label status. Overall, 87% of all patients across all cohorts expressed the importance of the drug’s availability as hugely or very important.

**Conclusion:**

The survey responses highlight the activity of sirolimus for EHE and the importance of securing a label extension for the drug delivering equitable access to this treatment for patients.

## Background

Epithelioid hemangioendothelioma (EHE) is an ultra-rare vascular sarcoma, with an incidence of 0.038/100,000/year and a prevalence of <1/1,000,000, originating from the endothelium of blood vessels. It can arise anywhere in the body including soft tissue, bones, visceral organs, muscles, and skin (1–5). It is reported that more than half of patients present with metastatic disease at diagnosis, most commonly involving liver, lung, pleura, and bones. EHE can occur at any age but is most prevalent between 30 and 50 years and is marginally more prevalent in women (5).

EHE is characterized predominantly by two chromosomal translocations leading in about 90% of cases to the *WW Domain Containing Transcription Regulator 1 (WWTR1) -* also called *transcriptional coactivator with PDZ-binding motif (TAZ) –* and *Calmodulin Binding Transcription Activator 1 (CAMTA1)* fusion gene, and approximately 10% to the *Yes-associated protein 1 (YAP1) and Transcription Factor Binding To IGHM Enhancer 3 (TFE3)* fusion gene (1, 5–8).

The clinical presentation can vary widely from indolent, asymptomatic disease to aggressive, symptomatic disease with widespread systemic involvement. The aggressive form acts as a high-grade sarcoma often with symptoms (pain, loss of weight, fatigue, and fever) and involves the lung or abdominal serosal surfaces (3, 9). There is currently no way to predict when indolent disease may become more aggressive, making any form of accurate prognosis impossible. As a result, the five-year survival expectancy ranges between 20% - 70% (10, 11).

No active medical treatments are specifically approved for EHE, while soft tissue sarcoma treatments are typically reported to be inactive (12). For localized disease, surgery or other locoregional treatments are the standard approach, including liver transplantation that is usually followed by immunosuppressive therapy to avoid organ rejection, often including mTOR inhibitors such as sirolimus. For asymptomatic patients with metastatic disease, active surveillance is commonly used as a first approach (3). For patients with disease progression and/or worsening of symptoms, systemic treatment is indicated. Local procedures are also sometimes an option to reduce disease burden/symptoms.

Systemic treatments are highly variable and rely largely on historical case reports and clinician experience. Patients are treated with soft tissue sarcoma treatment schedules, while other treatments shown to be active through clinical use are also used in clinical studies or ‘off label’ (12–21), but only if off-label use is feasible (based on insurance, health system or country-level medical regulations).

In 2020, clinical experts and patient advocates convened under the umbrella of the European Society for Medical Oncology (ESMO) to establish a consensus position on the management of EHE (3). The experts recognized that mTOR inhibitors represent the best treatment option in this setting (3), including sirolimus. mTOR inhibitors are not formally approved for treatment of sarcomas. However, retrospective data indicate sirolimus has antitumor activity in advanced and moderately progressive disease (16–21), with a growing body of real-world data supporting its use. However, with little interest from pharmaceutical companies in developing this class of compounds in EHE, access to sirolimus represents a major challenge as some patients are unable to receive the treatment off-label, resulting in inequalities in disease management and patient care. It is for this reason that repurposing of sirolimus for the treatment of EHE represents a solution to an unmet medical need.

Drug repurposing is a development strategy that seeks to establish new medical indications for existing licensed medications rather than from the *de novo* development of new molecules (22). Repurposing is particularly important for ultra-rare diseases where the low incidence, a lack of historical studies, limited research or commercial interest means it is impossible to generate data sets to comply with established regulatory procedures that are the standard for more common diseases. In October 2021, following the European Commission’s Expert Group on Safe and Timely Access to Medicines for Patients (STAMP) program, and recognizing these challenges, the EMA launched a pilot program to support the repurposing of medicines. The repurposing of sirolimus for progressive EHE was one of the projects selected for inclusion in the pilot, which has led to a continuing dialogue between regulators, clinicians, researchers, not-for-profit organizations, and patient advocates.

To complement dialogue with regulators and increase the available evidence, EHE patient advocates conducted a survey within the global EHE community to gain patients’ perspectives on sirolimus. This undertaking was primarily driven by The EHE Foundation (EHEF), from the USA, and the UK-based the EHE Rare Cancer Charity UK (EHERCC). The EHEF is a US-based non-profit 501c3 organization, founded in 2015, dedicated to pursuing effective treatments for EHE and supporting patients and their families. The Foundation serves the global EHE community with its mission to find treatments and a cure, by advancing research and driving collaboration between patients, researchers, and clinicians. The EHERCC was established in 2015 by the EHE patient community that had come together under the EHE Patient Support Facebook group. The charity has three core objectives: (i) patient support and advocacy; (ii) fundraising; and (iii) promoting and funding a dynamic research programme. EHERCC is an independent entity but works in close collaboration with other EHE foundations and patients’ groups established by the EHE patient community from 2015. While based in the UK, EHERCC is also engaged with and establishing, a pan-European patient network.

Notably, at the time of survey development, a formal EHE patient registry was not available. Results of the survey and conclusions that can be drawn are the subject of this manuscript.

## Materials and methods

### Survey development

The survey developers, as representatives of the patient community, conducted a thorough review of patients’ comments, questions, and experiences about the use of sirolimus shared in a private online Facebook community of ~2500 EHE members and/or carers to identify themes for development of a survey.

The survey was constructed using the online SurveyMonkey® platform. Questions were developed by EHE patient advocates. The question set is included in the **Supplementary Information**. An explanatory introduction of the survey was included to inform the targeted community of the rationale for the survey, as well as disclose the intended use of the data. Participation in the survey was voluntary, and participants were informed that their responses would be used anonymously for the purpose of bringing patients’ collective experiences to physicians and regulators. A privacy statement was published for participants. Participants were able to give their contact information and permission to be re-contacted by the survey administrators. Proceeding to answer the questions in the survey was interpreted as consent. As the inclusion of pediatric patients or patients in poor health might be expected, prospective participants who may be care givers for patients were instructed on how to answer questions about the patient. The survey was provided in English-language only.

### Survey design

**Figure 1:**
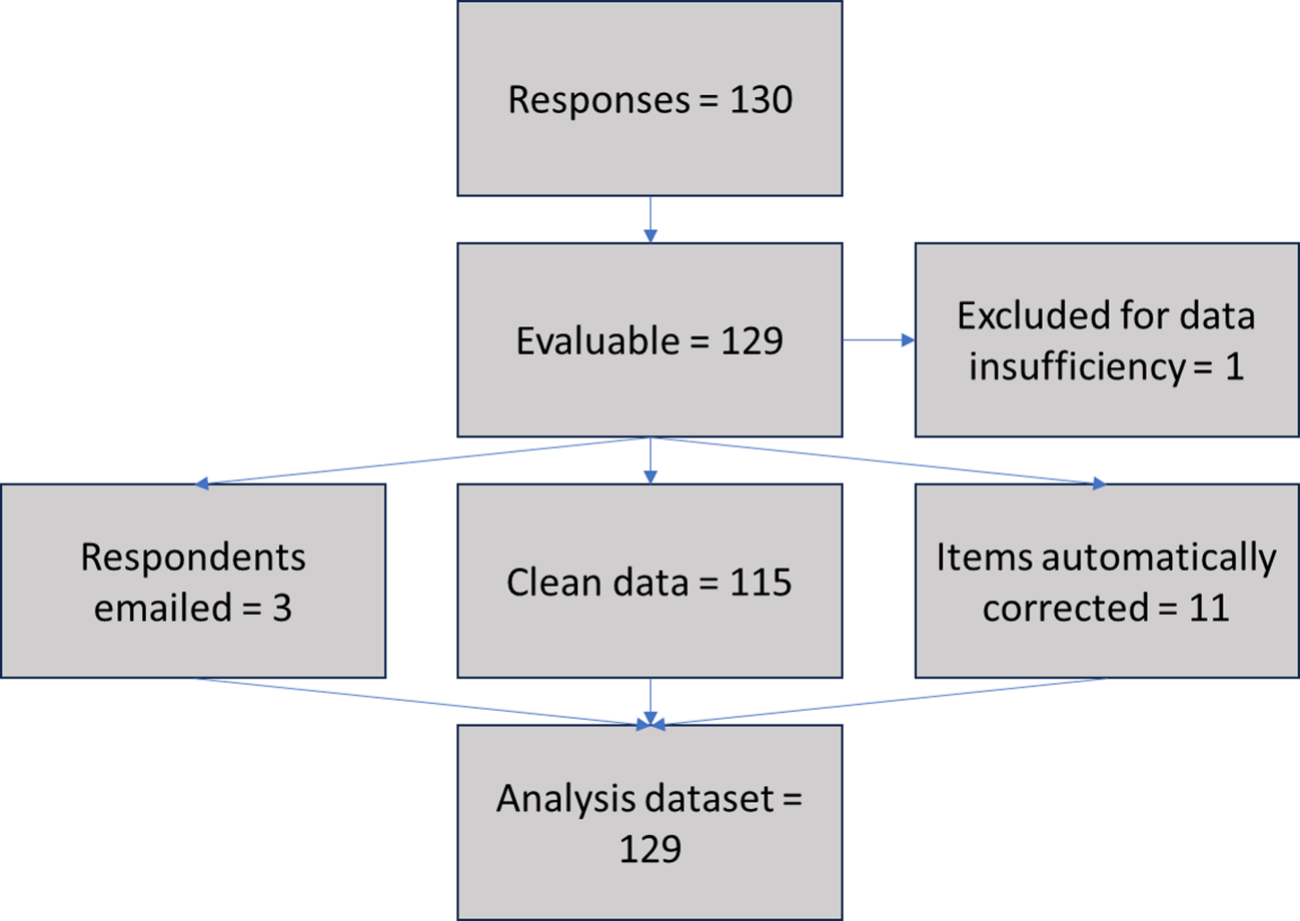
Analysis dataset construction workflow.

The survey consisted of 20 questions and sought to gain perspectives from all EHE patients on sirolimus, regardless of whether they had used the drug or not. The survey design did not define any exclusion criteria. Participants were asked to include their country of origin, year of birth and disease presentation. All participants had to enter the year of EHE diagnosis. Questions were developed with branching logic established based upon patients’ responses to key questions such as ‘*having had an organ transplant*’ (to identify patients taking sirolimus to avoid organ rejection following liver transplantation) or ‘*having ever taken or currently taking sirolimus*’. Responses to these initial questions ensured that participants would be asked questions relevant to their disease and treatment experiences. Evaluable responses were defined by inclusion of country, year of diagnosis, disease sites, liver transplant status, and sirolimus status (taking/have taken or never taken). The survey branching logic is shown in **Figure 1** of the **Supplementary Information**.

### Survey distribution and administration

The survey was introduced to the global EHE community via email and social media channels. The EHEF and EHERCC maintain email subscriber lists for communication with their constituents; 698 patients and caregivers were emailed with appropriate follow-up emails over a 14-day period. Additionally, the survey was publicized widely in the private Facebook group. Advocacy groups in Australia, Canada, and Italy similarly shared in their regions. The survey did not permit more than one response from the same device to reduce the risk of participants responding more than once.

Data were collected and stored by EHEF and EHERCC, with results presented in de-identified, aggregate form to the EHE community and regulators. The raw data were downloaded as an Excel formatted table, with one row of data per participant. An automated process was developed to take the raw data and cleanse and standardize it, with a clear audit trail of changes generated by this process.

### Statistical methods

We performed a descriptive analysis based on the results of the survey. Microsoft Excel 365 64-bit (version 2310) was used for all statistical analysis and the generation of descriptive statistics. Age was calculated as the year of birth subtracted from 2023, with missing values excluded. Years of disease was calculated as the year of diagnosis subtracted from 2023, with missing values excluded. All charts and tables were generated using Microsoft Excel.

For analysis, participants were classified as belonging to one of three groups: the sirolimus group (Group S), this includes all non-liver transplant patients taking or having taken sirolimus for EHE; and the non-sirolimus group (Group NS), this includes all non-liver transplant patients not taking or have never taken sirolimus; and the liver transplant group (Group LT), this includes all liver transplant patients, irrespective of current or past treatment with sirolimus. Liver transplant patients were treated as a separate cohort as they may be taking sirolimus, other mTOR inhibitors or other immune suppresants for prophylaxis against organ transplant rejection. The effect of sirolimus in this patient population would be difficult to interpret.

## Results

### Description of patient groups

From 11/02/23 to 26/02/23, a total of 130 responses from individual EHE patients or carers from 21 countries worldwide were recorded. 129/130 (99%) were considered adequate for the present analysis (1/130 contained only two completed fields and was excluded for data insufficiency). 115/129 (89%) of the evaluable surveys did not require data correction, while 11/129 (9%) included fields that could be automatically corrected (i.e., date formats standardized), 3/129 (2%) required follow up via email and 2/129 (1%) required text translation. The data flow for the data cleansing and standardization process is shown in **Figure 1**.

### Description of participants

Of 129 participants, 32/129 (25%) were included in Group S, 76/129 (59%) in Group NS, and 21/129 (16%) in Group LT, **Table 1**. The median age (years) of all participants was 47.6 (range 14-81); in Group S, 39.4 (range 14-74), Group NS 51.1 (range 15-81) and Group LT 47.4 (range 32-71). Overall, participants had a median 6.6 years since EHE diagnosis (range 1-26), 7.4 (range 1-26) for Group S, 6.1 (range 1-23) for Group NS, and 9.4 (range 2-21) for Group LT.

**Table 1:** Demographic and disease presentation information by patient cohort.

	All	Group S (Sirolimus)	Group NS (Non-sirolimus)	Group LT (Liver transplant)
Number of participants	129	32 (24.8%)	76 (58.9%)	21 (16.3%)
Age (Years)				
Mean	47.6	39.4	51.1	47.4
Range	14 - 81	14 - 74	16 - 81	32 - 71
SD	15.5	18.0	13.4	14.1
Years of disease				
Mean (Years)	6.6	6.1	6.0	9.4
Range (Years)	1 - 26	1 - 26	1 - 23	2 - 21
SD (Years)	5.6	5.2	5.5	6.0
Disease Presentation at time of the survey				
Liver only	24 (19%)	2 (6%)	14 (18%)	8 (38%)
Bone only	0 (0%)	0 (0%)	0 (0%)	0 (0%)
Lung only	7 (5%)	2 (6%)	5 (7%)	0 (0%)
Pleura only	1 (1%)	1 (3%)	0 (0%)	0 (0%)
Other only	8 (6%)	0 (0%)	8 (11%)	0 (0%)
Liver/lung	34 (26%)	7 (22%)	18 (24%)	9 (43%)
Liver/lung/bone	9 (7%)	2 (6%)	6 (8%)	1 (5%)
Liver/lung/bone/other	12 (9%)	8 (25%)	4 (5%)	0 (0%)
Liver/lung/other	11 (9%)	2 (6%)	7 (9%)	2 (10%)
Liver/bone	3 (2%)	0 (0%)	2 (3%)	1 (5%)
Liver/lungs/bones/pleura/other	1 (1%)	1 (3%)	0 (0%)	0 (0%)
Lung/bone	6 (5%)	2 (6%)	4 (5%)	0 (0%)
Lung/pleura	4 (3%)	1 (3%)	3 (4%)	0 (0%)
Lung/bone/other	1 (1%)	0 (0%)	1 (1%)	0 (0%)
Lung/other	7 (5%)	3 (9%)	4 (5%)	0 (0%)
Bone/other	1 (1%)	1 (3%)	0 (0%)	0 (0%)
Total	129 (100%)	32 (100%)	76 (100%)	21 (100%)

**Figure 2:**
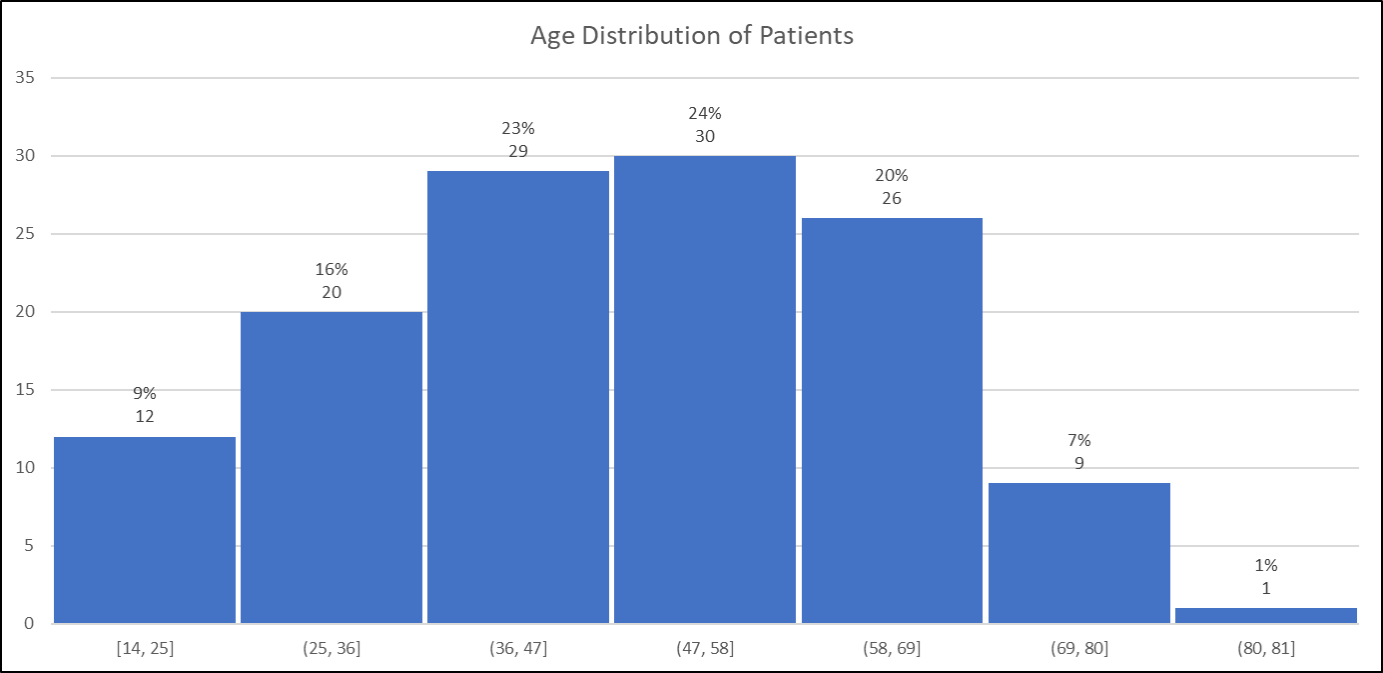
Distribution by age of participants.

**Figure 2** shows the distribution by (decadal) age of participants, note that 2/129 participants did not include year of birth. The distribution shows no indications of age-related bias.

The highest number of responses were from United States (n=50/129, 39%), followed by United Kingdom (n=19/129, 15%), Canada (n=12/129, 9%), Italy (n=9/129, 9%), and Australia (n=9/129, 7%). Number of responses by country is shown in **Supplementary Table 1**.

### Disease presentation

Reported disease presentation is shown in **Table 1**, both overall and by patient group.

### Patients’ experiences and reported responses in Group S

**Figure 3:**
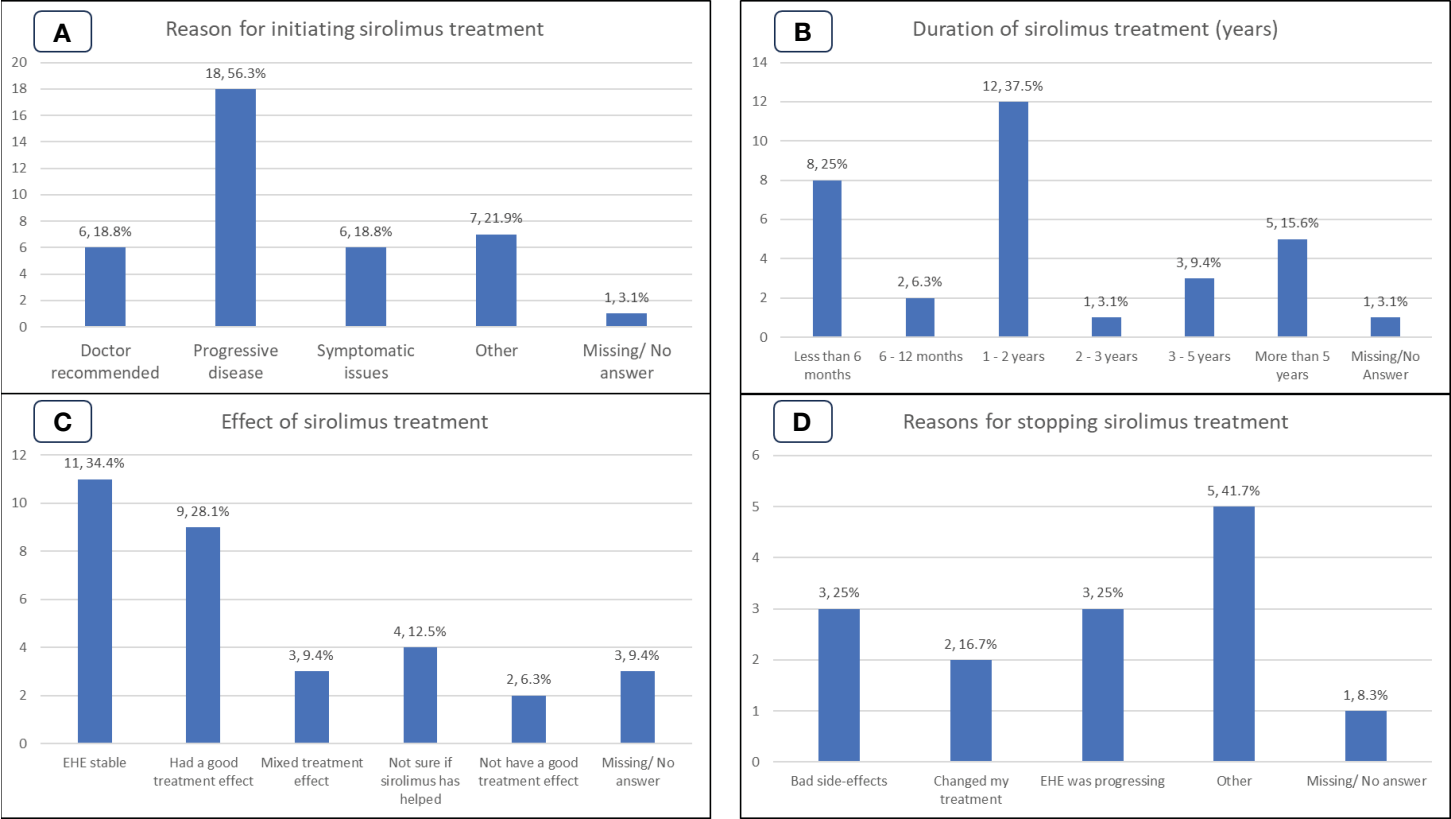
Patient experiences of the Group S (sirolimus) cohort. **(A)**: Reasons for initiating sirolimus treatment. **(B)**: Duration, in years, of sirolimus treatment. **(C)**: Patient perspective on the effect of sirolimus treatment. **(D)**: Reasons for cessation of sirolimus treatment.

Group S contained 32/129 (25%) participants. **Figure 3A** shows the reasons for sirolimus treatment initiation. The most common reason was ‘progressive disease’ (n=18/32, 56%); 6/32 (19%) responded ‘my doctor recommended’, 6/32 (19%) responded ‘had significant EHE-related symptoms’. Six/32 (19%) patients included more than one reason as per the survey structure.

At the time the survey was undertaken 20/32 (63%) patients were still on treatment with sirolimus, while 12/32 (37%) had discontinued therapy. Reasons for discontinuation, shown in **Figure 3D**, were side effects (3/12, 25%); disease progression (3/12, 25%); clinician decision (2/12, 17%), other (2/12, 16%). Other reasons cited by patients included stopping sirolimus to take part in a clinical trial (1/12, 8%) and lack of symptomatic relief (1/12, 8%). Of the 11 patients who selected a reason for discontinuation, 5/12 (42%) could be directly attributed to the drug not working. Note that 2/12 (17%) patients reported multiple reasons for stopping sirolimus.

The duration of sirolimus treatment is shown in **Figure 3B**. Duration of treatment was available for 31/32 (97%) patients and ranged from ‘less than 6 months’ to ‘more than 5 years. Participants reporting duration of treatment as ‘less than 6 months’ (n=8/32, 25%) or ‘6-12 months’ (n=2/32, 6%) while durations of more than 1 year were (n=21/32, 66%). Of note, 5/32 (16%) participants reported duration of treatment for more than 5 years and 16/32 (50%) participants reported duration of 1-5 years treatment.

When asked what effect treatment with sirolimus was having/had had, 11/32 (34%) reported disease stabilization; 9/32 (28%) tumors shrank, or tumors stopped growing, 3/32 (9%) mixed treatment effect on tumors (some tumor lesions shrank, some did not shrink or some stopped growing and remained stable, while some grew), 2/32 (6%) reported not having a good treatment effect on tumors or EHE-related symptoms indicating disease progression, 4/32 (13%) were unsure if sirolimus had helped their EHE, as shown in **Figure 3C**.

**Figure 4:**
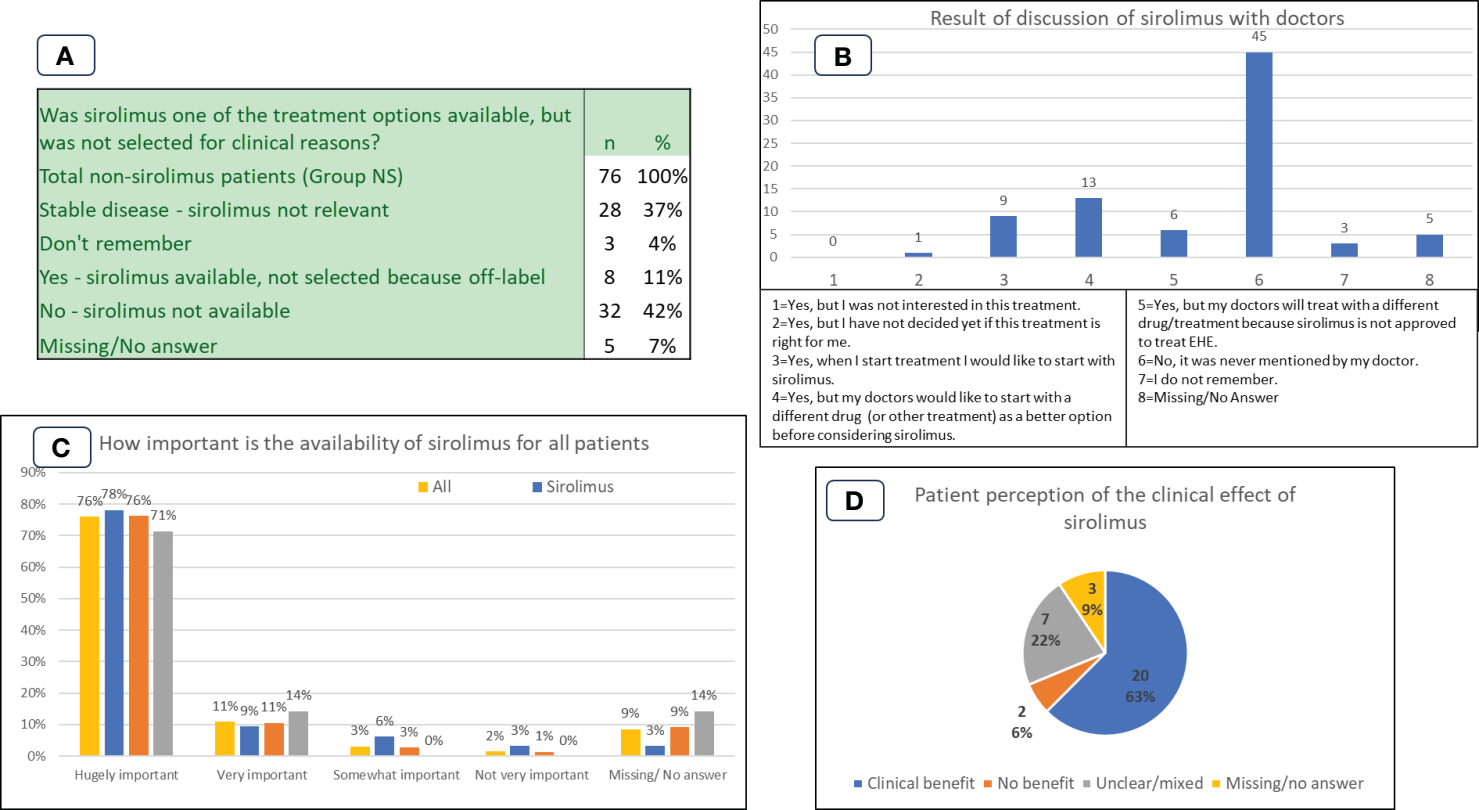
Patient experiences and perspectives in Group NS (non-sirolimus) and Group S (sirolimus) cohorts. **(A)**: Availability of sirolimus as a treatment option. **(B)**: Results of discussion of sirolimus with doctors. **(C)**: Importance of availability of sirolimus for all patient cohorts. **(D)**: Group S patient perception of the clinical effect of sirolimus.

Patients’ self-reported outcomes for Group S are shown in **Figure 4D**. If we combine the stable and good treatment effect outcomes as patient perception of clinical benefit, 20/32 (63%) of the group regarded the treatment as of benefit, as shown in **Figure 4D**. Only 2/32 (6%) patients perceived that the treatment had no clear benefit, and 7/32 (22%) reported mixed outcomes.

### Patients’ experiences and perspectives in Group NS

Group NS included 76/129 (59%) participants.

### Availability of sirolimus to patients

Participants in the Group NS were asked ‘Was sirolimus one of the treatment options available, but was not selected for clinical reasons?’ The results are shown in **Figure 4A**. 40/76 (53%) replied yes or no, the rest either having stable disease (28/76), not remembering (3/76), or not answering (5/76). For 32/76 (42%) participants sirolimus was not available, and for 8/76 (11%) sirolimus was available but not selected for treatment. A related question asked whether sirolimus was available but was not selected because it was not approved for EHE, i.e., it would have to be used off-label. Of the 8 patients who answered that it was available but not selected in **Figure 4A**, all (100%) responded that it was due to being off-label.

### Discussion of sirolimus with doctors

Patients in the NS group were asked if sirolimus was ever discussed or mentioned as a treatment option by their doctor. Results are shown in **Figure 4B**. Participants were given the option to select more than one response.

### Importance of access and availability of sirolimus to all participants

Participants were asked to select how important it is to have sirolimus available to all EHE patients. Scoring ranged from ‘hugely important’ to ‘not very important’, as shown in **Figure 4C**. The question conceivably covers all the uses of sirolimus – including for progressive disease and for prophylaxis against organ transplant rejection, but the high importance reported by patients is consistently high across all groups. 98/129 (76%) participants from all three participant groups placed availability of sirolimus to all patients as ‘hugely important’.

## Discussion

This survey, conducted by EHE patient advocates within the global EHE patient community to gain patients’ perspectives on sirolimus for EHE, involved 129 participants from 21 countries, and successfully achieved its primary objective of gaining deeper insights into the treatment experiences and perspectives of patients related to this systemic therapy. Considering the patient perception of clinical benefit resulting in 20/32 (63%) of participants reporting clinical benefit, the survey findings further endorse the consensus position of the ESMO community of sarcoma experts, suggesting that the mTOR inhibitor sirolimus should be regarded as a front-line treatment for progressive EHE.

To the best of our knowledge, this is the first dataset produced within the patient community, yielding important knowledge from a real-world setting of such a rare disease. This effort provides valuable information from a patient cohort, contributing significantly to our understanding of the management of the disease and patient experiences.

The outreach approaches to ask EHE patients to participate in the survey via relevant social media channels, email, and newsletters, ensured that the risk of bias was reduced by not limiting by region or disease-specific sub-groups. The participation of patients from 21 countries worldwide further ensured the representative nature of the participant cohort and the reduction of the risk of skewing of results due to a single national jurisdiction or health system. The inclusion of a majority of participants (64%) having never taken sirolimus for any reason also demonstrates that there was no unintentional bias with, for example, just sirolimus patients participating.

The objective of demonstrating that the survey patient cohort was representative of EHE in terms of the age distribution of the patients was also met with a good comparison of characteristics as described in the literature for EHE (10). Disease presentation reported by participants aligns closely with the most reported presentations of EHE in literature, which include liver, lung, and bones (10). Participants who have taken or are still taking sirolimus reported very similar EHE presentation as those who have not taken sirolimus.

To keep the survey appropriately simple for participants, we included two measures of potential efficacy that could be answered by patients with limited medical or scientific knowledge. The first was the duration that they had been on the drug, and the second was a prescribed menu of effects the drug had had on their EHE.

Duration on the drug is seen as a good indicator as clinicians only start to prescribe systemic treatments when they have verified disease progression, through growing tumor burden on radiological imaging and/or increasing symptoms. The effect of the systemic drug prescribed is then monitored over a period of typically 3 to 6 months, with dosage increases often tried if disease progression continues. In the event of the drug failing to have a positive impact, patients will be moved to another treatment. Patients who were still on the drug were therefore requested to confirm how long they had been taking sirolimus. Any duration of more than 1 year is viewed as positive, and multi-year durations are seen as exceptional when compared against reported outcomes of other drugs within the EHE patient community. In our sample of patients taking sirolimus as a systemic treatment for EHE, 66% of patients have been taking sirolimus for more than 12 months, and 29% had been taking it for more than 2 years. Five patients, 16%, had been on sirolimus for more than five years.

We wanted to understand the main reason for the cessation of treatment. A simple menu of reasons for stopping treatment was provided. A category of ‘other’ with the ability to provide additional reasons in open text was included to allow for reasons not included in the prescribed answers. Of the 32 participants in the sirolimus group, 12 (38%) had ceased treatment with the drug. The clearest lack of efficacy signal is progressive disease, which was the reason listed by 3 participants, representing 9% of the sirolimus group. Some reasons for cessation appear to be unrelated to the drug efficacy, for example participating in a clinical trial, using a treatment protocol limited to two years of sirolimus, or misdiagnosis of disease progression. Three patients discontinued due to side effects, and one patient commented that the drug did not provide symptomatic relief.

Overall, the rate of discontinuation does not appear to be entirely driven by lack of efficacy, even though this is difficult to confirm from the survey.

The importance of having sirolimus available for EHE patients is underscored by the data in **Figure 4C**. The overwhelming response to this question is either ‘hugely important’ (76%) or ‘very important’ (11%). It is notable that the answers do not differ by patient group. The data from **Figure 4A** show that for some patients the question is pressing. For 32 (42%) patients the drug is not available because it would have to be used off-label, and for 8 (11%) it is available but has not been prescribed by treating physicians due to the drug not being approved for EHE in the specific country. **Figure 4B** illustrates the absence of dialogue about sirolimus as a treatment option, reiterating the importance of education and advocacy to ensure patients and their clinicians are well-informed about efficacious treatment options, including off-label therapies.

Overall, while providing a valuable data set in such a rare disease, we acknowledge that this survey has limitations. These include the lack of detailed treatment history, limited description of disease presentation, symptoms assessments, insurance or health care payment requirements, and information about where participants receive care. We recognize that an opportunity was missed in not including biological gender in the survey that would have provided a further valid parameter for demonstrating that the participant cohort was representative of the disease.

We also recognize that the survey does not collate more detailed knowledge of a participant’s disease, nor the specific developments that led to the decision to start systemic treatment. There is also no record of whether a participant was prescribed sirolimus as the first-line treatment for their progressive EHE, or whether other drugs had been prescribed and failed prior to taking sirolimus. This additional information would provide a more detailed description of each patient’s situation but was felt to run the risk of overwhelming patients resulting in their non-participation. For this reason, the relatively simple survey described above was adopted.

However, we believe that these data, that provide patients’ perspectives, add to what is already available in the literature and are aligned with results available from retrospective studies of sirolimus in EHE. They can further inform researchers, clinicians, and regulators and should be included in future discussion on sirolimus in this tumor type and in future surveys and PROs assessment.

Since this survey, the EHE Global Patient Registry (https://fightehe.org/registry/), a natural history study of EHE, has been ethically approved and is enrolling EHE patients from all countries to gather additional data about treatment with sirolimus, and all other disease management therapies and therapeutic strategies. Further analysis and publications of these invaluable data are anticipated in the future.

## Conclusions

An EHE diagnosis presents an unpredictable journey, for doctors and patients, resulting in uncertainty in both prognosis and future treatments, and high levels of patient anxiety, while those who are living with advanced or progressive disease face significant unmet medical needs. For such patients, it is reasonable to expect that they and their clinicians want all options available that can keep the disease stable by deterring or halting growth of tumors and symptoms.

Faced with a devastating EHE diagnosis, patients need hope beyond a natural desire to survive. For patients, the hope of an effective treatment option has surfaced with growing data from the clinical and patient community regarding the positive outcomes of treatment with sirolimus. Data collected in this survey demonstrates further that people living with EHE who have taken or are currently taking sirolimus are experiencing benefit. These results also justify mTOR inhibitors being selected by the ESMO community of experts to be the front-line systemic treatment for the disease, and why the drug is now being prescribed off-label in many major jurisdictions. Patients are also aware that sirolimus has been approved by multiple regulatory bodies for the treatment of other indications, including prophylaxis against organ transplant rejection and lymphangioleiomyomatosis. These approvals give assurance to patients that the drug is safe for consumption, with controlled dosing as indicated.

However, while data supports sirolimus as an efficacious systemic treatment for EHE, there are still patients who cannot access the drug while it is only available off-label. This inequitable access to an effective drug is unacceptable, and it is to remedy this situation that EHE patient advocates, with clinicians and researchers, are working with regulators to secure the necessary approvals so that patients may have access to and can benefit from sirolimus regardless of their location. Indeed, patients have a right to expect regulators to recognize the challenges faced by ultra-rare cancers and accept evidence appropriate to the rarity of the disease and expeditiously approve drugs that are proven to have safe, positive effects on the patients’ disease, quality of life, and life expectancy.

## Data availability statement

The raw data supporting the conclusions of this article will be made available by the authors, without undue reservation.

## Ethics statement

Ethical approval was not required for the studies involving humans because the data was from a patient survey, with details of how the data was to be used included in the survey. Completion of the survey indicated informed consent. No identifiers are used and the data is fully anonymized. The studies were conducted in accordance with the local legislation and institutional requirements. Written informed consent for participation was not required from the participants or the participants’ legal guardians/next of kin in accordance with the national legislation and institutional requirements because completion of the survey indicated informed consent.

## Author contributions

DR: Conceptualization, Data curation, Investigation, Methodology, Writing – original draft, Writing – review & editing. HL: Conceptualization, Data curation, Methodology, Validation, Writing – original draft, Writing – review & editing. GB: Formal analysis, Methodology, Validation, Writing – original draft, Writing – review & editing. WT: Formal analysis, Validation, Writing – review & editing. RJ: Formal analysis, Validation, Writing – review & editing. SS: Formal analysis, Validation, Writing – review & editing. PP: Data curation, Formal analysis, Investigation, Methodology, Validation, Writing – original draft, Writing – review & editing.
