# Supplementary material for: The patient perspective on sirolimus for epithelioid hemangioendothelioma (EHE): results of a community survey highlighting the importance of equitable access to treatments
Source: Front Oncol. 2024 Feb 26;14:1367237. doi: 10.3389/fonc.2024.1367237 (PMC10925709; doi:10.3389/fonc.2024.1367237)
Supplement: Supplementary file 1 [file DataSheet_1.docx]

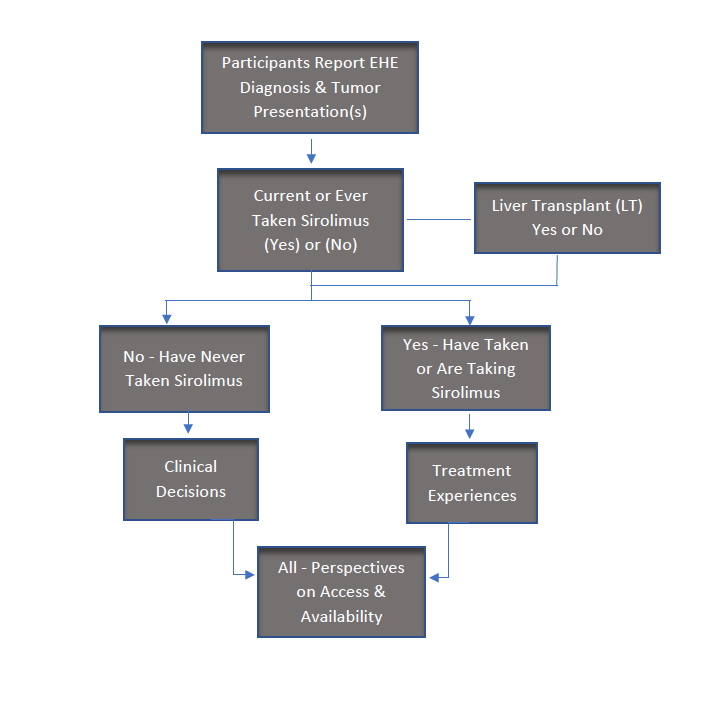


Figure 1 - Survey design and branching logic diagram

| Country | Liver Transplant | Sirolimus | Non-sirolimus | Country Total (%) |
| --- | --- | --- | --- | --- |
| Australia | 1 | 1 | 7 | 9 (7%) |
| Canada | 1 | 5 | 6 | 12 (9%) |
| Chile | 0 | 1 | 0 | 1 (1%) |
| Denmark | 0 | 1 | 1 | 2 (2%) |
| Estonia | 1 | 0 | 0 | 1 (1%) |
| France | 0 | 0 | 2 | 2 (2%) |
| Germany | 0 | 1 | 2 | 3 (2%) |
| Hungary | 0 | 0 | 1 | 1 (1%) |
| Israel | 0 | 1 | 0 | 1 (1%) |
| Italy | 2 | 6 | 1 | 9 (7%) |
| Netherlands | 0 | 0 | 2 | 2 (2%) |
| Poland | 3 | 0 | 1 | 4 (3%) |
| Portugal | 0 | 0 | 1 | 1 (1%) |
| Romania | 0 | 0 | 1 | 1 (1%) |
| Russia | 0 | 1 | 3 | 4 (3%) |
| Spain | 0 | 0 | 2 | 2 (2%) |
| Sweden | 0 | 0 | 1 | 1 (1%) |
| Switzerland | 0 | 1 | 2 | 3 (2%) |
| Ukraine | 0 | 1 | 0 | 1 (1%) |
| United Kingdom | 2 | 1 | 16 | 19 (15%) |
| USA | 11 | 12 | 27 | 50 (39%) |
| Total | **21** | 32 | 76 | 129 |

Table 1- Distribution of participants by country

**Patients’ Perspectives on Sirolimus for EHE – Full question set and supporting text**

Sirolimus is an established drug that has been used extensively for many years as an immunosuppressant after organ transplant, as well as a treatment for other diseases. In recent years, some doctors have been prescribing sirolimus to manage EHE. It does not work in all cases, but there is evidence that sirolimus can establish or re-establish stability in some EHE patients.

Currently, sirolimus is not approved for EHE. This means that it can only be prescribed ‘off label’, which may prevent some patients from getting access to this treatment because some doctors will not prescribe it because it is not approved, or it may not be funded by insurance companies or national health systems. The EHE foundations are working with regulators in several regions to advocate for the approval of sirolimus for EHE.

To support our effort, we want to hear from EHE patients in our global community who have ever considered sirolimus, have ever taken sirolimus, or would like to have the option to have sirolimus as a treatment option so that we can better describe patients’ perspectives and experiences to doctors and regulators. We have made a simple survey to gain your perspectives and experiences.

The information you provide is only intended to help us organize and bring your collective experiences to doctors and regulators. All information you provide is optional, and we will only contact you with your permission. All data will be kept private, and no data will be shared with any other party or included in any documentation other than in a completely anonymized manner.

We are very grateful for your time and the information you provide, if you choose to participate in this survey. If you have any questions, please contact xxx.

As you answer the questions in this survey please answer about the person who has EHE, in the case

that you are answering for someone you care for who has EHE.

Thank you for providing your answers! We hope that this effort will lead to greater access to sirolimus for all EHE patients.

- What is your year of birth?
- What country do you reside in?
- What year was your EHE diagnosed?

Where are your EHE tumors? *Select all that apply.*

- Liver
- Lung(s)
- Pleura (lining of lung)
- Bone(s)
- Muscle(s)
- Skin
- Vessels
- Other (please specify)

Have you had a liver transplant, or any other organ transplant?

- Yes
- No

Are you currently taking sirolimus, or have you ever taken sirolimus?

- Yes
- No
- I do not know

Was sirolimus one of the treatment options available, but was not selected for clinical reasons?

- Yes
- No
- I do not remember.
- I have not discussed treatment because my EHE is stable.

Was sirolimus not available to you as a treatment because it would have to be used off-label (not approved specifically for EHE)?

- Yes
- No
- I do not know

Was sirolimus ever discussed or mentioned as a treatment option by your doctor? *Please select all the options that best describe your situation.*

- Yes, but I was not interested in this treatment.
- Yes, but I have not decided yet if this treatment is right for me.
- Yes, when I start treatment I would like to start with sirolimus.
- Yes, but my doctors would like to start with a different drug (or other treatment) as a better option before considering sirolimus.
- Yes, but my doctors will treat with a different drug/treatment because sirolimus is not approved to treat EHE.
- No, it was never mentioned by my doctor.
- I do not remember.

Why did you start treatment with sirolimus? Please select the option(s) that best describe the reason. *You may select more than one.*

- My doctor recommended I take sirolimus, but I am not sure why.
- I had an organ transplant.
- My EHE was believed to be progressive as I had tumour growth over a period of time.
- I had significant EHE-related symptoms (e.g., pain, weight loss, fatigue, fever, fluid on lungs or in abdomen).
- Other (please specify)

Approximately how long have you been taking, or did you take sirolimus? *Please select the option that best matches your treatment.*

- Less than 6 months
- 6 - 12 months
- 1 - 2 years
- 2 - 3 years
- 3 - 5 years
- More than 5 years

What effect has (or did) sirolimus had / have on your EHE? *Select the option that best describes your treatment response.*

- It has kept my EHE stable, with no growth, no new tumors or only very slowly growing.
- It had / has had a good treatment effect on my tumours (my tumours shrank, or my tumours stopped growing).
- It did not have a good treatment effect on tumors or symptoms (the disease continued to progress).
- It had a mixed treatment effect on tumours (some tumours shrank, and some did not shrink; some tumours stopped growing and some remained stable, some grew).
- I am not sure if sirolimus has helped my EHE.

Are you still taking sirolimus

- Yes
- No

If you have stopped taking sirolimus, why did you stop this treatment? *Select all that apply.*

- My EHE was progressing
- I had bad side-effects
- I / my doctor changed my treatment plan; wanted/needed to have more aggressive or targeted treatment
- I am still taking sirolimus
- Other (please specify)

Recognizing that sirolimus is not effective for all EHE patients, how important is it to you to know that you have access to sirolimus as a treatment for your EHE if you and your doctor choose this therapy?

How important is it to you that sirolimus is available **to all EHE patients** as a treatment option, if they and their doctor choose this treatment?

- **Hugely important:** having a treatment available to me gives me real hope that my EHE can possibly be stabilized and that gives me comfort to believe that I can have a full life.
- **Very important:** it does give me a much more positive outlook on treatment options, even knowing that it may not work for every person.
- **Somewhat important:** it does not hurt but, I do not know if it improves the chance of survival or long term disease control, but it might.
- **Not very important:** it may benefit some people, but it may not benefit many people.
- **Not important:** sirolimus may stabilize EHE for a while, but I am interested in treatments that improve the longer-term outlook.

Do you have any other comments or experiences related to sirolimus that you would like to share?

The EHE Group, as collaborators on this survey, would like to have your permission to contact you to get additional feedback from you about sirolimus, or to keep you updated as we continue discussions with doctors and regulatory authorities about this drug.

- Yes, you can keep my contact information and contact me in the future.
- No, please do not contact me in the future.
- What is your first name? *(optional)*
- Email address for contact:

**Thank you very much for providing your experiences and perspectives about sirolimus and for completing these questions.**
